# Extracellular Matrix Composition and Remodeling: Current Perspectives on Secondary Palate Formation, Cleft Lip/Palate, and Palatal Reconstruction

**DOI:** 10.3389/fcell.2019.00340

**Published:** 2019-12-13

**Authors:** Katiúcia Batista Silva Paiva, Clara Soeiro Maas, Pâmella Monique dos Santos, José Mauro Granjeiro, Ariadne Letra

**Affiliations:** ^1^Laboratory of Extracellular Matrix Biology and Cellular Interaction, Department of Anatomy, Institute of Biomedical Sciences, University of São Paulo, São Paulo, Brazil; ^2^Clinical Research Laboratory in Dentistry, Federal Fluminense University, Niterói, Brazil; ^3^Directory of Life Sciences Applied Metrology, National Institute of Metrology, Quality and Technology, Duque de Caxias, Brazil; ^4^Center for Craniofacial Research, UTHealth School of Dentistry at Houston, Houston, TX, United States; ^5^Pediatric Research Center, UTHealth McGovern Medical School, Houston, TX, United States; ^6^Department of Diagnostic and Biomedical Sciences, UTHealth School of Dentistry at Houston, Houston, TX, United States

**Keywords:** palatogenesis, extracellular matrix, extracellular matrix remodeling, metalloproteinases, cleft lip/palate, palatal reconstruction, tissue bioengineering, biomaterials

## Abstract

Craniofacial development comprises a complex process in humans in which failures or disturbances frequently lead to congenital anomalies. Cleft lip with/without palate (CL/P) is a common congenital anomaly that occurs due to variations in craniofacial development genes, and may occur as part of a syndrome, or more commonly in isolated forms (non-syndromic). The etiology of CL/P is multifactorial with genes, environmental factors, and their potential interactions contributing to the condition. Rehabilitation of CL/P patients requires a multidisciplinary team to perform the multiple surgical, dental, and psychological interventions required throughout the patient’s life. Despite progress, lip/palatal reconstruction is still a major treatment challenge. Genetic mutations and polymorphisms in several genes, including extracellular matrix (ECM) genes, soluble factors, and enzymes responsible for ECM remodeling (e.g., metalloproteinases), have been suggested to play a role in the etiology of CL/P; hence, these may be considered likely targets for the development of new preventive and/or therapeutic strategies. In this context, investigations are being conducted on new therapeutic approaches based on tissue bioengineering, associating stem cells with biomaterials, signaling molecules, and innovative technologies. In this review, we discuss the role of genes involved in ECM composition and remodeling during secondary palate formation and pathogenesis and genetic etiology of CL/P. We also discuss potential therapeutic approaches using bioactive molecules and principles of tissue bioengineering for state-of-the-art CL/P repair and palatal reconstruction.

## Introduction

The first studies on palate development and cleft lip/palate (CL/P) date back to the beginning of the 20th century (Whitehead, 1902; Fawcett, 1906; Tweedie, 1910). These studies were fundamental to our understanding of the molecular and cellular aspects that drive palate formation, that when disrupted, may explain the occurrence of CL/P. CL/P is the most common craniofacial anomaly occurring in approximately 1 in 700 live births, and representing a substantial burden worldwide (Shaw, 2004; Massenburg et al., 2016). The treatment of this disorder is complex and demands a multiplicity of health professionals to perform numerous interventions throughout the patient’s life (Kantar et al., 2019). Besides the high cost of treatment, CL/P imposes a significant impact on the quality of life of affected children and their families (Macho et al., 2017; Sundell et al., 2017). The primary treatment for CL/P repair is surgical correction, frequently including autologous bone grafts from the iliac crest to repair the palatal bone defect. This increases hospitalization time, pain, and donor site morbidity; hence, new strategies for the use of regenerative therapies and bone graft substitutes are needed to reduce the morbidity associated with the condition and improve treatment outcomes and patient’s quality of life (Sharif et al., 2016). Further, the identification of key factors involved in the etiology of CL/P may provide the foundation for the development of bioactive molecules and precision therapy approaches for CL/P.

Extracellular matrix (ECM) genes, soluble factors, and enzymes responsible for ECM remodeling (e.g., metalloproteinases) are expressed during lip and palate development and suggested to play a role in the etiology of CL/P. However, a comprehensive evaluation of ECM dynamics during palatogenesis is still fragmented. Historically, the ECM was considered to be the scaffold that provided an adequate architecture for tissue structure. Subsequently, knowledge of the soluble factors secreted by the cells into the ECM and its function as storage site for the rapid bioavailability of several molecules demonstrated the role of ECM as a crucial component of the cellular microenvironment (Ricard-Blum and Vallet, 2016, 2019). An intricate balance between proteases that degrade the ECM components and their inhibitors maintain the ECM homeostasis. Abnormal ECM remodeling (excessive or inefficient) is often involved in the development and progression of several pathologies and conditions, including CL/P.

This review focuses on the role of genes involved in ECM composition and remodeling during secondary palate formation and with regard to the genetic etiology of CL/P. It also presents an overview of therapeutic approaches using bioactive molecules and principles of tissue bioengineering for state-of-the-art CL/P repair and palatal reconstruction.

## Craniofacial Development and Palatogenesis

Embryonic development is a precise temporal and spatial multistep process that is coordinated by specific molecular patterns, cell–cell and reciprocal cell–ECM interactions from the totipotent stem cell up to a fully developed organism. The vertebrate craniofacial complex arises from three embryonic tissue layers (endoderm, mesoderm, and ectoderm) and multipotent migrating neural crest cells (NCCs), also known as the “fourth layer.” NCCs are a population of epithelial cells within the neural tube, which migrate and then undergo epithelial–mesenchymal transition (EMT) prior to neural tube closure, delaminating from the neuroepithelium, and migrating toward the growing swellings. NCCs contribute to neural structures found in the whole vertebrate body and ectomesenchyme of the head and neck and originate the mesenchymal/stromal stem cells (MSCs)/progenitors that will differentiate into the dermis, skeletal, and connective tissues of the face and the neck, being the primary source of mesenchymal tissue in this region. They are also responsible for the bones and cartilage of the head and neck, while the trunk and appendicular members come from the mesoderm. Craniofacial development is one of the most complex processes in an organism and one-third of birth defects arise from errors in this process, causing significant infant mortality (Weston and Thiery, 2015; Francis-west and Crespo-Enriquez, 2016; Liu and Cheung, 2016; Dupin et al., 2018; Pla and Monsoro-Burq, 2018; Rothstein et al., 2018).

Lip and palate formation occurs in a series of coordinated steps, which take place between the fourth and 12th gestational week (GW) in humans and between the 11th and 15.5th embryonic day (ED) in mice. Facial development begins by frontonasal (central), maxillary, and mandibular (laterals) prominences growing around the primitive oral cavity, called the stomodeum, to give rise to the face. NCCs from distinct sites of the developing brain, such as the midbrain and forebrain cells (frontonasal), midbrain and hindbrain cells (maxillary), where the mix of the midbrain and hindbrain cells and mesenchyme from the first pharyngeal arch (mandibular) enrich these prominences (Jankowski and Márquez, 2016).

The frontonasal prominence is the most fundamental structure for external nose and the primary palate formation. Between the fifth–seventh GW and 10.0th–11.0th ED, it expands from two ectoderm nasal or olfactory placodes which each enlarge to divide into the nasomedial and nasolateral processes. The nasomedial processes grow downward and merge to originate the globular or intermaxillary process, which will form the philtrum of the upper lip and primary palate. Anatomically, the primary palate is anterior to the incisive foramen and contains the maxillary incisors (Jankowski and Márquez, 2016).

**FIGURE 1:**
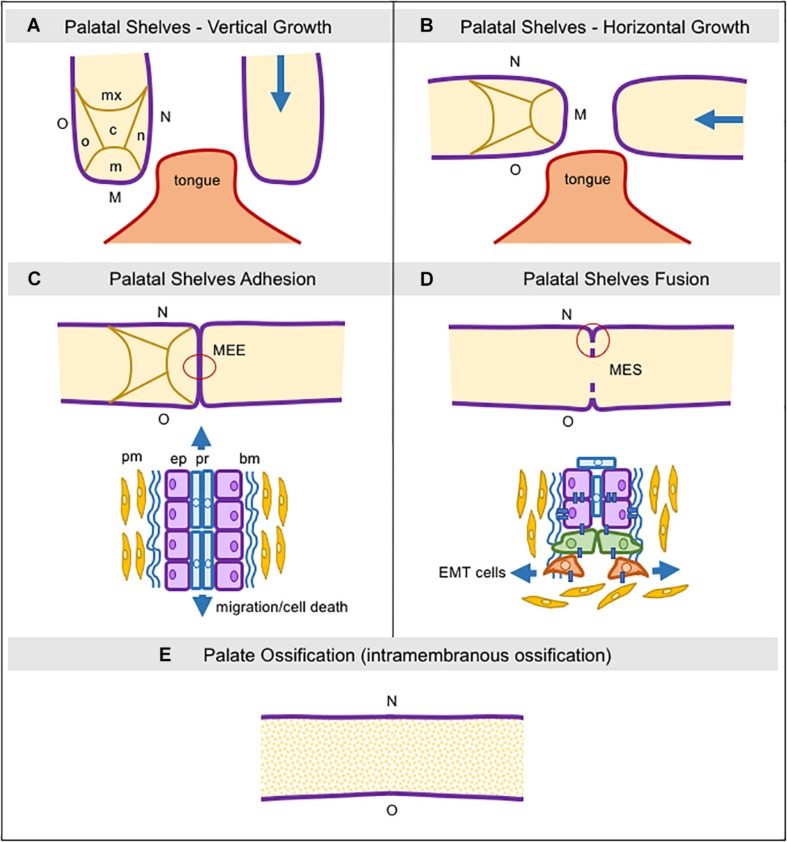
Schematic representation of consecutive steps during secondary palatogenesis. **(A)** Initially, palatal shelves grow down, surrounding the tongue, and five regions in the palatal mesenchyme can be seen: nasal, medial, oral, central, and maxillary, as well as three regions in the palatal epithelium: nasal, medial, and oral. **(B)** Palatal shelve orientation switches from vertical to horizontal, toward each other and above the tongue. **(C)** The medial palatal epithelia from both shelves establish adhesion and it is now named the Media Edge Epithelia (MEE). In this area, we can see two different types of epithelial cells localized in two layers, the peridermal and the non-stratified cuboid epithelium. Peridermal cells start to migrate toward both nasal and oral epithelial sites. **(D)** The MEE starts the fragmentation since the epithelial layer begins the epithelial-to-mesenchymal transition and these cells then migrate into the palatal mesenchyme. **(E)** The MES completely disappears and the palatal mesenchymal cells start to differentiate into osteoblasts via intramembranous ossification. bm: basement membrane; c: central region; EMT: epithelial-to-mesenchymal transition; ep: epithelial cells; M: medial site; m: medial region; MEE: medial edge epithelia; MES: medial epithelial seam; mx: maxillary region; n: nasal region; N: nasal site; o: oral region; O: oral site; pm: palatal mesenchyme; pr: peridermal cells.

The maxillary prominences drive the formation of the upper part of the face, lip, upper jaw (maxillae), and the secondary palate. The mandibular prominences originate the lower part of the face, lip, and lower jaw (mandible). Briefly, the secondary palate develops from two mesenchymal projections (palatal shelves) derived from maxillary prominences extending inferiorly and bilaterally to the tongue (Figure 1A). With the progressive development of the mandible, these projections assume a horizontal position above the tongue (Figure 1B; Jankowski and Márquez, 2016). Subsequently, the adhesion of the epithelium from both palatal shelves forms a single line, called medial edge epithelia (MEE), which must disappear to allow for palatal tissue confluence and fusion (Figure 1C). Programmed cell death, EMT, and cell migration from the oral to the nasal epithelia, or a combination of these events have been suggested as plausible mechanisms for MEE disintegration, albeit this remains a controversial issue (Figure 1D; Ray and Niswander, 2012; Hammond et al., 2017).

Once palatal fusion is complete, the anterior two-thirds mineralize by intramembranous ossification (hard palate) (Figure 1E), and the posterior third will give rise to a fibromuscular tissue (soft palate) under the signaling by numerous factors, particularly bone morphogenetic proteins (BMPs), fibroblast growth factors (FGFs), hedgehog (HH), vascular endothelial growth factor (VEGF), and Wnt/β-catenin signaling, which drive the palatal mesenchyme to undergo osteoblast differentiation for mineralization (Wu et al., 2008; Baek et al., 2011; Nelson et al., 2011; Pan et al., 2013; Smith et al., 2013; Hill et al., 2014, 2015; Nassif et al., 2014; Iyyanar and Nazarali, 2017; Zhang et al., 2017; Xu J. et al., 2018; Thompson et al., 2019).

Anatomically, the fusion of primary and secondary palates with the nasal septum originates the palate, a physical barrier that separates the fully developed nasal and oral cavities. Physiologically, it has a function in breathing, speech, and swallowing. The local regulation of palate development depends on a network of several factors, such as transcription factors, signaling molecules, soluble factors, ECM proteins, ECM remodeling enzymes, ECM cross-linkers, and cell adhesion molecules. Disturbance of this tightly controlled network may inhibit the fusion of the palatal shelves and, hence, result in a cleft palate (Funato, 2015).

## ECM Structural Molecules and Soluble Factors

**FIGURE 2:**
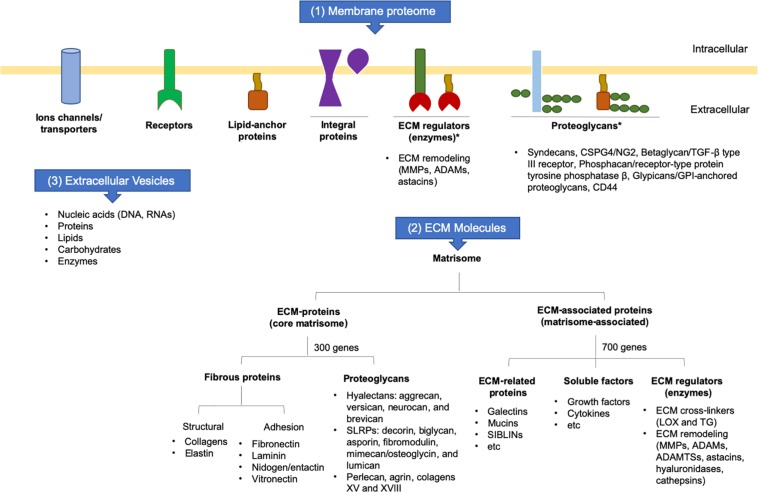
Schematic representation of cell surface molecules and secretome. ADAM: adisintegrin and metalloproteinase; ADAMTS: adisintegrin and metalloproteinase with thrombospondin motifs; ECM: extracellular matrix; GPI: glycosylphosphatidylinositol; LOX: lysyl oxidase; MMP: matrix metalloproteinase; SIBLIN: small integrin-binding ligand *n*-linked glycoprotein; SLRP: small leucine-rich proteoglycans; TG: transglutaminase; TGF-: transforming growth factor beta.

Collectively, the secretome is the set of membrane proteins that are tethered on the cell surface and interact with the ECM, secreting molecules into ECM in soluble forms or inside of extracellular vesicles (EVs). Part of the secretome contains the matrisome, which encompasses all ECM-proteins and ECM-associated proteins. The core matrisome is composed mainly of structural proteins encoded by around 300 genes, whereas matrisome-associated proteins are those that modulate ECM functions and are encoded by about 700 genes, corresponding to 4% of the human or mouse genomes. The increasing knowledge regarding specific ECM tissue signatures contributes to the understanding of the role of the ECM in development, homeostasis, tissue repair, and disease (Hynes and Naba, 2012; Figure 2).

Fibrous proteins and proteoglycans are the two principal components of the core matrisome. Fibrous proteins are responsible for the matrisome’s supportive function (collagen and elastin) and adhesive functions (fibronectin, laminin, nidogen, and vitronectin). These macromolecules interact with each other and can binding numerous growth factors (Raghunathan et al., 2019).

Proteoglycans are proteins conjugated to GAG chains and are crucial for conferring resistance to compression forces. Most GAGs are highly negatively-charged molecules that attract positively-charged sodium ions and, consequently, water, contributing to the viscosity and hydration within tissues. Among GAGs, chondroitin, dermatan, heparan, and keratan sulfate are the principal GAGs associated with proteoglycans in the connective tissues. High levels of hyaluronic acid or hyaluronan (HA), a non-sulfated GAG, are also found in the ECM (Garantziotis and Savani, 2019). Depending on the type of GAG associated with the proteoglycan, this will determine its location in the cytoplasm (the only member is serglycin), on the cell surface or within the ECM. Most of the heparan sulfate proteoglycans are anchored on the cell membrane via their transmembrane domains or glycosylphosphatidylinositol (GPI) anchors. Thus, proteoglycans can interact with many other molecules, including ECM remodeling enzymes and growth factors, thereby playing an important role in regulating ECM dynamics (Iozzo and Schaefer, 2015).

The provisional matrix is a transitory ECM found during the early stages of development, tissue repair, and disease which is later replaced by a tissue-specific ECM. It is formed by fibrin, fibrinogen, fibronectin, HA, and versican, a large chondroitin sulfate proteoglycan. HA provides a “glue” core to bind all other components and place it in the pericellular space, due to its interaction with a specific membrane receptor, CD44. Several growth factors can stimulate the expression of these macromolecules. The provisional matrix has a viscoelastic property that allows it to create spaces within the ECM, providing means for cell migration. For example, the migratory routes of NCCs during early embryonic development highly express high levels of versican (Barker and Engler, 2017; Chester and Brown, 2017; Wight, 2017) and tenascin (Riou et al., 1992). It is well known that collagens type I, III, IV, V, VI, fibronectin, HSPG, laminin, and tenascin are expressed during palatogenesis *in vivo* (Ferguson, 1988; Dixon et al., 1993a) and EGF or TGF-α can stimulate their expression on mouse embryonic palatal mesenchymal cells *in vitro* (Dixon et al., 1993b). The intrinsic “internal shelf force” for palatal elevation has been attributed to HA since it is the most abundant GAG in palatal ECM before shelf elevation (Ferguson, 1988). It is produced on the cell membrane surface by specific enzymes (HA synthases—Has 1-3) and these are differentially expressed in palatal mesenchyme and epithelium during palatogenesis (Galloway et al., 2013). In TGFβ-3 null mice, expression of all Has forms is decreased, leading to reduced amounts of HA and impaired shelf elevation (Galloway et al., 2013). Recently, Has2 has been described to be a crucial HA synthase in NCC-derived mesenchyme during craniofacial development and palatogenesis (Lan et al., 2019). Also, FGFs induce HA synthesis by mouse embryonic palatal mesenchymal cells *in vitro* (Sharpe et al., 1993). Fibronectin is found during embryonic development in areas characterized by cell migration (Schwarzbauer and DeSimone, 2011). It appears that fibronectin arrangement is vital for cell migration and palatal shelf elevation. In this case, Rac1 and cell density modulates fibronectin deposition in mid-palate (Tang et al., 2015). Moreover, Rac1 is downregulated by retinoic acid, leading to the cleft palate as a consequence of the disarrangement of fibronectin and cell migration as well (Tang et al., 2016).

Cellular communication is a well-known mechanism in which cells can communicate with each other and modify cell behavior through soluble factors. Intercellular communications occur via direct cellular interactions in which cell surface proteins act as mediators able, or not, to bind to the ECM (juxtacrine signaling). Alternatively, cells release local mediators into the ECM to create self-control signals (autocrine signaling) and send information to neighboring cells (paracrine signaling) or reach target cells in long distances via hormones (endocrine signaling) (Ansorge and Pompe, 2018). The local mediators are peptides or growth factors which control many cellular activities. During development, a combination of cell–cell interactions occurs, as well as the secretion of mediators named morphogens, which induce specific cell differentiation in a distinct spatial order and morphogen gradient-dependent manner (Inomata, 2017). The main morphogens are retinoic acid, HH, TGF-β, BMPs, and Wnt/β-catenin.

The actions of numerous morphogens in palatogenesis have been extensively studied, mainly secreted factors such as HH (Cobourne and Green, 2012; Dworkin et al., 2016; Xavier et al., 2016; Li et al., 2018), FGF (Jiang et al., 2006; Nie et al., 2006; Snyder-Warwick and Perlyn, 2012; Stanier and Pauws, 2012; Prochazkova et al., 2018; Weng et al., 2018), TGF-β (Nawshad et al., 2004; Iwata et al., 2011; Nakajima et al., 2018), BMP (Nie et al., 2006; Parada and Chai, 2012; Graf et al., 2016), and Wnt/β-catenin family proteins (He and Chen, 2012), which are responsible for guiding all steps of palate formation by reciprocal signaling between the embryonic oral epithelium and palatal mesenchyme, as well as transcription factor regulation (Greene and Pisano, 2010; Levi et al., 2011; Bush and Jiang, 2012; Li et al., 2017). Also, other morphogens and growth factors have emerged in palatogenesis, such as connective tissue growth factor (Tarr et al., 2018) and retinoic acid (Okano et al., 2014; Mammadova et al., 2016). Dysregulation of these pathways through genetic variations in individual genes has been suggested as strongly associated with CL/P (Pauws and Stanier, 2007; Krejci et al., 2009; Tang et al., 2013; Okano et al., 2014; Reynolds et al., 2019).

During the last decade, knowledge of new types of RNA with regulatory functions, located in non-coding regions of DNA, has improved our understanding of gene expression regulation (Scherrer, 2018). Many different microRNAs have been identified to temporally and spatially regulate morphogens and transcription factors during palatogenesis (Eberhart et al., 2008; Seelan et al., 2014; Ding et al., 2016; Reiss and Bhakdi, 2017; Schoen et al., 2017). Not surprisingly, microRNAs have been suggested as be new targets for investigating in CL/P studies (Li et al., 2010; Wang et al., 2013, 2017; Ma et al., 2014; Gao et al., 2015; Li D. et al., 2016; Li J. et al., 2016; Schoen et al., 2017, 2018; Chen et al., 2018; Grassia et al., 2018; Pan et al., 2018; Suzuki et al., 2018; Wu N. et al., 2018; Xu M. et al., 2018).

## ECM Remodeling

The extracellular microenvironment is dynamically modeled and remodeled by soluble or EV-associated proteases secreted into the ECM or membrane-anchored proteases, which are classified as cross-linkers and remodeling proteases (Sanderson et al., 2019). Of the ECM cross-linkers, lysyl oxidases (LOX) and transglutaminases (TGs) are the major families responsible for establishing cross-links between the central core matrisome proteins. Moreover, an intricate balance between proteases and their inhibitors maintains the ECM homeostasis; abnormal ECM remodeling (excessive or inefficient) is involved in the development and/or progression of several pathologies due to modifications in macromolecule composition (posttranscriptional control and posttranslational modifications), biophysical (architecture), and biomechanical properties (stiffness).

### ECM Cross-Linkers

**FIGURE 3:**
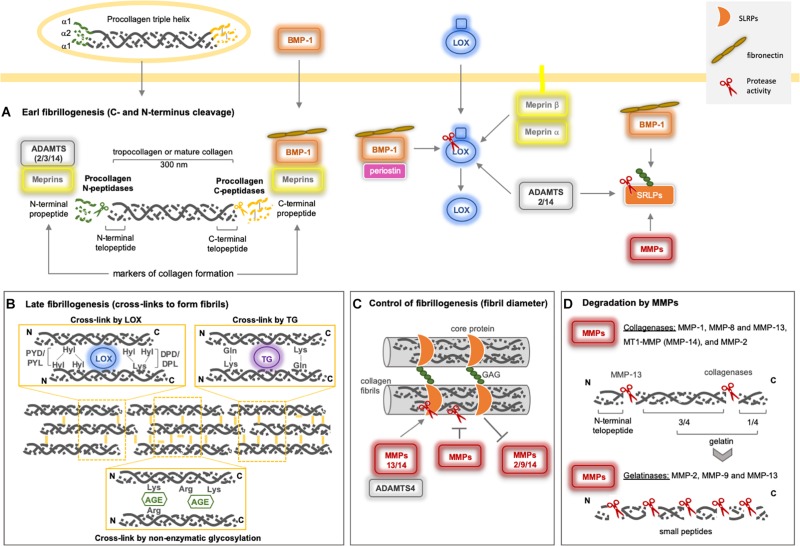
Collagen type I assembly and degradation by metalloproteinases. **(A)** The early fibrillogenesis process on fibrillar procollagens starts by the excision of both the N- and C- termini by classical Procollagen N-peptidases (sub-group of the ADAMTS family) and Procollagen C-peptidases (BMP-1). They cleave procollagens I, II, III, and V (ADAMTS2/14 and BMP-1), where ADAMTS3 can cleave only procollagen II. Recently, both meprins have been reported to be able to remove both N- and C- terminus as well. **(B)** After N- and C- termini removal from procollagen, mature collagen is the target for ECM cross-linker enzymes (LOX and TG), which will form collagen fibrils. Under high glucose conditions, a non-enzymatic cross-link takes place, named AGE. **(C)** The level of collagen fibrillogenesisis controlled by proteoglycans (SRLPs), which can interact with collagen fibrils, resulting in the modification of the diameter of collagen fibers. Fibromodulin, decorin, and lumican prevent or delay the cleavage of collagen by MMP-1 and MMP-13. Lumican acts on MMP-14 enzymatic inhibition (Pietraszek et al., 2014) and gene expression (Niewiarowska et al., 2011; Malinowski et al., 2012). Decorin inhibits MMP-2 and MMP-9 gene expression and activity (Neill et al., 2012). MMP-14 can cleave human decorin (Mimura et al., 2009) and thelumican core (Li et al., 2004), MMP-13 and ADAMTS-4 also cleave decorin (Shu et al., 2019). **(D)** Among the metalloproteinases, only MMPs can cleave fibrillar collagens. The collagenases generate ¼ and 3/4 fragments (gelatin) that are further cleaved by gelatinases (MMPs-2 and -9) and MMP-13. MMP-13 also removes the N-terminal telopeptide from collagen. Indirectly, metalloproteinases may modulate collagen fibrillogenesis by processing LOX and SRLPs. BMP-1 and ADAMTS2/14 activate proLOX in distinct sites and seem to be essential for LOX-collagen binding (Rosell-García et al., 2019). Periostin increases the proteolytic action of BMP-1 on proLOX (Maruhashi et al., 2010). BMP-1 promotes the maturation of SLRPs (von Marschall and Fisher, 2010), as well as the MMPs, after C-terminal excision. Fibronectin increases BMP-1 activity against biglycan and procollagen I (Huang et al., 2009). AGE: advanced glycation end products; ADAM: adisintegrin and metalloproteinase; ADAMTS: adisintegrin and metalloproteinase with thrombospondin motifs; LOX: lysyl oxidase; MMP: matrix metalloproteinase; SLRP: small leucine-rich proteoglycans; TG: transglutaminase.

Post-translational modifications (cross-links) in collagen–collagen, collagen–ECM, and ECM–ECM interactions are relevant for the integrity, stiffness, and rigidity of the microenvironment. Once formed, cross-links formed are immature but become more stable due to multivalent cross-links that generate insoluble protein polymers that are resistant to proteolytic degradation, improving the biomechanical properties of the collagen network. Procollagen maturation takes place when both N- and C-termini are classically removed by ADAMTS and BMP-1/TLD (BTPs), respectively, but this cleavage may also be mediated by meprins, forming tropocollagen (Figure 3A). Subsequently, three main pathways promote the final fibrillogenesis: the LOX-mediated, TG-mediated, and sugar-mediated cross-linking pathways (Figure 3B). The latter pathway constitutes a non-enzymatic glycosylation reaction that occurs as the consequence of prolonged exposure to reducing sugars (e.g., ribose and glucose), producing advanced glycation end products (AGEs), which are associated with aging and diabetic complications (Benkovics et al., 2017; Cruz et al., 2018).

The LOX and LOX-like (1–4) proteins are a family of copper-dependent amine oxidase enzymes that catalyze the formation of unstable aldehydes by the oxidation of the ε-amino groups of lysine/hydroxylysine in collagens and lysine in elastin, forming covalent cross-linkages in collagen–collagen and collagen–elastin complexes, respectively. LOX is secreted as a proenzyme into the ECM, but is also found intracellularly, and is then processed by BMP-1, the same enzyme that cleaves fibrillar procollagens (Figure 3). As such, there is a direct relationship between the collagen process and its cross-linking, suggesting a major role for LOX in ECM orientation (Rodriguez-Pascual and Rosell-Garcia, 2018).

The TGs [formed of nine members, including tissue TG (tTG) or TG2, which is the most abundant in tissues] belong to a multi-functional family of calcium-dependent acyl-transferase enzymes that catalyze transamidation of glutamine and lysine to form covalent bonds both inside and outside of the cell. These cross-links form between collagen–collagen, collagen–ECM, or ECM–ECM and can involve fibronectin, mainly, and also nidogen/entactin, osteonectin, osteopontin, laminin, vitronectin, fibrinogen, and heparan sulfate proteoglycan. Initially, the TG catalyzes the formation of an isopeptide bond between y-carboxamide groups of glutamine residue side chains and the ε-amino groups of lysine residue side chains with subsequent release of ammonia. Subsequently, glutamine and lysine residues are available to bind with peptides or proteins, and the intra- or inter-molecular ε-(γ-glutamyl)lysine cross-links take place. In the presence of water, TGs are also able to hydrolyze glutamine residues to glutamic acid by deamidation (Figure 3B). These cross-links exhibit high resistance to physical, chemical, and proteolytic degradation, mainly by matrix metalloproteinases (MMPs). Physiologically, TGs generate biological polymers that are indispensable for creating barriers and stable structures in several systems, while pathologically, they contribute to generating fibrotic matrices (Eckert et al., 2014; Savoca et al., 2018).

### ECM Remodeling Enzymes: Metalloproteinases

Most of the ECM remodeling enzymes belong to the metzincin family (Stöcker et al., 1995), which share numerous similarities, including multiple domains, zinc-dependent endopeptidases, a typical structural profile and tertiary configurations of the catalytic domain (the secondary structure contains three histidines bound to zinc, at the catalytic site, and a methionine, or “Met-turn”). This family comprehends vertebrate matrixins (MMPs), adamalysins (ADAMs—a family of disintegrin and metalloproteinase, mainly sheddases; and ADAMTSs—a family of disintegrins and metalloproteinases with thrombospondin-like motifs, mainly formed of proteoglycanases and procollagen N-propeptidases), astacins (BMP-1/Tolloid-like protease 1 and Meprins, mainly formed of procollagen N- and C-propeptidases), and pappalysins (main bioavailability of IGFs) (Cerdà-Costa and Gomis-Rüth, 2014) and encodes around 200 genes, identified in humans and mice, comprising around 1/3 of proteases, the largest proteolytic enzyme group existent (López-Otín and Bond, 2008). Among them, MMPs are classically recognized to degrade all ECM components, but other metalloproteinases have been recognized to play essential roles in ECM maturation and to generate bioactive molecules. As a result of the extensive study of ECM remodeling enzymes over the last six decades, other biological functions have also been attributed to them, due to their broad spectrum of substrates, identified in both subcellular and extracellular compartments (Bond, 2019). Other enzymes, such as urokinase-type plasminogen activator, cathepsins, and heparanases, are also indispensable in these processes, and growth factor bioavailability within the ECM is protease-dependent. As a result of the extensive study of ECM remodeling enzymes over the last six decades, other biological functions have also been attributed to metalloproteinases, due to their broad spectrum of substrates, identified in both subcellular and extracellular compartments (Bond, 2019). We will focus only on metalloproteinases currently known to have a role in palatogenesis and CL/P.

Among metzincins, ADAMs, ADAMTSs, and MMPs are closely related in structure, regulation, and activation. However, they have different substrates and, therefore, distinct functions under physiological and pathological conditions. Structurally, the N-terminal ectodomain of most secreted MMPs is composed of pre-, pro-, and catalytic domains (metalloproteinase domain) and contains a furin region in all ADAM, ADAMTS, and membrane-anchorage MMPs. Complementary domains confer proteolytic specificity and localization. As such, MMPs are the most studied metalloproteinases and act in many cellular functions (e.g., proliferation, migration, differentiation, among others) due to their cleavage of substrates in the ECM, on the cell surface and intracellularly (cytoplasm and nucleus) to produce bioactive molecules. However, few studies have been conducted to understand the interrelationship between these metalloproteinases and how they work together to control cell behavior.

#### MMP Family

Over the years, the “degradative” activity of MMPs during physiological and pathological processes has led to their association with tissue destruction, due to their unique ability to cleave fibrillar collagens (Sprangers and Everts, 2019). However, “omic” studies and a better understanding of ECM dynamics support a broader role for MMPs in pathological and physiological events (Rodríguez et al., 2010). Several other core matrisome, ECM-associated proteins, and cell surface proteins, cleaved by MMPs, reveal hidden sequences and unblock the diverse cell functions (Butler and Overall, 2009; Deryugina and Quigley, 2010; Bauvois, 2012; Mannello and Medda, 2012).

Extensive reviews focus on MMPs regulation at both intra and extracellular levels and have been extensively reviewed. At the gene transcription level, signals from the ECM (cytokines, growth factors, EMMPRIN/ECM metalloproteinase inducer/CD147, integrins, ECM proteins, cellular stress, morphological changes, among others) significantly impact MMP expression. Mutations and polymorphisms in MMP genes (particularly in promoter regions), together with epigenetic modifications, have been shown to modulate MMP expression. Post-transcriptional regulation includes changes in mRNA stability through microRNAs, decoy RNAs, and degradation pathways. MMPs are targets of several PTMs that are crucial for correct cellular localization (via insertion of the GPI-anchor), intracellular activation of membrane-anchoring MMPs by furins, and insertion of carbohydrates (*N* and *O*-glycosylation) (Boyd, 2006; Reuben and Cheung, 2006; Vincenti, 2007; Clark et al., 2008).

In the ECM, soluble proenzymes are activated by other proteinases, mainly active MMPs, via the “cysteine switch mechanism.” The catalytic activity may be inhibited by their endogenous inhibitors in the ECM (TIMPs) or on the cell membrane (RECK). Additionally, their proteolytic activity may be modulated by allosteric control in exosites outside of the catalytic site and also by interactions with proteoglycans and GAGs (Pietraszek-Gremplewicz et al., 2019). For example, MMP-2 interacts with syndecan-2 on the cell surface, which blocks the activation of proMMP-2. In some cases, proenzymes are associated with TIMP (low concentration) for correct activation; for example, the MMP-14/TIMP-2/proMMP-2 ternary complex. MMPs may be associated with other ECM components, such as proteoglycans and GAGs, leading to their specific localization in the cellular perimeter or at ECM sites distant from the cell secretion point (Van den Steen et al., 2001; Hernandez-Barrantes et al., 2002; Ra and Parks, 2007; Gabison et al., 2009; Hadler-Olsen et al., 2011; Piperi and Papavassiliou, 2012; Rietz and Spiers, 2012; Li et al., 2014; Arpino et al., 2015; Karamanos et al., 2019).

Due to their unique ability to cleave fibrillar collagens, collagenases MMP-1, -8, and -13 are the main enzymes for collagen turnover and generate gelatin (classical ¾ and ¼ fragments) (Figure 3D). MMP-2 and -9, along with membrane-anchored MMPs (MMP-14 and MMP-16), may also cleave fibrillar collagen with different affinities, as is the case of collagen I and III, which are preferentially cleaved by MMP-1, whereas collagen II is the preferred substrate for MMP-13. Additionally, stromelysins (MMP-3 and -10) can degrade just collagen III, but not collagen I or II (Sprangers and Everts, 2019). The interaction of the N-terminal site of specific SRLPs (decorin, fibromodulin, and lumican) with collagen fibrils forms a layer that can prevent or delay the collagen cleavage by MMPs (Geng et al., 2006). At the same time, different SRLPs are substrates or inhibitors of MMPs and ADAMTS (Shu et al., 2019; Figure 3C).

#### ADAMTS Family

N-glycosylation, O-fucosylation, and C-mannosylation are the most frequent post-translational modifications (located at the ancillary domains) to control ADAMTS activity. These modifications control secretion, localization, activation, and catalytic functions. Cleavage of the central thrombospondin type 1 sequence repeat (TSR—probably attached to the cell membrane) regulates both the proteinase activity and the localization of these enzymes. The proenzymes can be activated intracellularly, on the cell surface or within ECM by furin-like pro-protein convertases and then autocatalytic activation. Similarly to ADAMs, ADAMTSs are selectively inhibited by TIMPs, where TIMP-3 is the principal inhibitor (Bekhouche and Colige, 2015; Kelwick et al., 2015; Dancevic et al., 2016).

Although ADAMTS-like homologues lack protease domains, ancillary domains are present and may be involved in the regulation of ADAMTS due to competitive binding to substrates. Furthermore, they may have ADAMTS-independent functions in ECM, cell–EMC, or cell–cell interactions. Recently, an unexpected interaction between LOX-ADAMTSLs was reported, suggesting a role in microfibril assembly (Aviram et al., 2019). Mutations or deletions in ADAMTS have been implicated in many pathologies and syndromes, where they modulate tissue morphogenesis and remodeling, in cancer, inflammation, in the central nervous system, and in cartilage and vascular biology (Kintakas and McCulloch, 2011; Stanton et al., 2011; Lisi et al., 2014; Dubail and Apte, 2015; Hubmacher and Apte, 2015; Kelwick et al., 2015; Rodríguez-Manzaneque et al., 2015; Dancevic et al., 2016; Fu and Kong, 2017; Itoh, 2017; Lemarchant et al., 2017; Yang et al., 2017; Mead and Apte, 2018).

## The Roles of MMPsand TIMPs in Palatogenesis

The development of the facial primordia requires remodeling of the ECM, which is mediated in part by MMPs. During embryonic development, the process of morphogenesis involves MMP-mediated changes in the composition of the ECM that further allow for cell migration and differentiation, cell–cell interactions, and tissue resorption. MMPs act on ECM remodeling during palatal shelves orientation and during EMT events for palatal fusion (Brown et al., 2002).

Early studies in mice have provided biological evidence for the roles of MMPs and TIMPs in embryonic development (Gack et al., 1995; Iamaroon et al., 1996; Blavier and DeClerck, 1997). Several MMPs, TIMPs, and RECK mRNA and proteins are expressed, in association with enzymatic activity, throughout the stages of murine palate development. These expressions share the same specific spatial and temporal distribution patterns in areas in which their preferred substrates are also present (Mansell et al., 2000; Morris-Wiman et al., 2000a, b; Blavier et al., 2001; Brown and Nazarali, 2010; de Oliveira Demarchi et al., 2010; Gkantidis et al., 2012).

**FIGURE 4:**
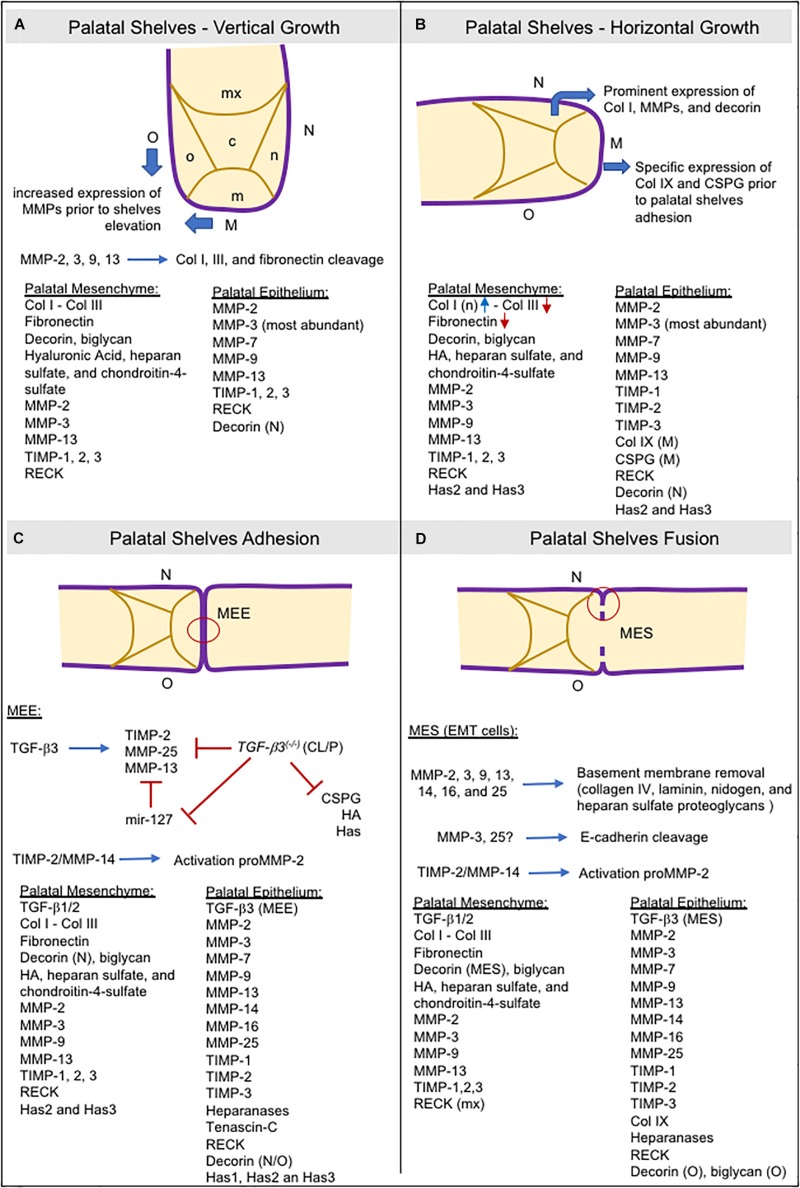
Schematic representation of consecutive steps during secondary palatogenesis and the molecules expressed in palatal mesenchyme ECM and palatal epithelium. Collagens type I and III, fibronectin, GAGs (hyaluronic acid, heparan sulfate, and chondroitin-4-sulfate), proteoglycans (biglycan, decorin), and TGF-β are the most abundant ECM molecules found in provisional ECM during palatogenesis. MMPs are the major protein responsible for ECM remodeling in both the ECM mesenchyme and basement membrane, but also expressing heparanases as well. Expression of TIMPs and RECK occurred, and they are crucial for ECM remodeling balance. **(A)** Most ECM components are widely present in palatal mesenchyme. MMP expression is high in the oral and medial epithelia before shelve elevation. **(B)** The increased expression of collagen I and decorin in the nasal region of the palatal mesenchyme may help with palatal elevation. Decorin can bind to collagen I and cause contraction of collagen *in vitro*. In the medial epithelium, the specific expression of collagen IX and CSPG is vital for adequate palatal shelve adhesion. **(C)** TGF-β3 modulates the expressions of TIMP-2, MMP-13, MMP-25, and CSPG in the MEE. TGF-β3 knockout mice downregulated these genes. mir-127 is upregulated, leading to repression of MMP-13 expression (Warner et al., 2015). **(D)** Other MMPs are required for MES disruption and are involved in basement membrane degradation by EMT cells. CSPG: chondroitin sulfate proteoglycans; EMT: epithelial-to-mesenchymal transition; HA: hyaluronic acid; Has: hyaluronan synthases; M: medial site; m: medial region; MEE: medial edge epithelia; MES: medial epithelial seam;mx: maxillary region; n: nasal region; N: nasal site; o: oral region; O: oral site; TGF-β: transforming growth factor beta.

The secretion of GAGs in the palatal mesenchyme is related to an increase in water content, and the specific accumulation of collagen I in the nasal side of the palatal mesenchyme may be necessary to generate internal forces required for shelf elevation. Furthermore, MMP expression increases in both the medial and oral epithelium before shelf elevation (Morris-Wiman et al., 2000a; de Oliveira Demarchi et al., 2010), as does MMP gelatinolytic activity in the basement membrane and beneath the mesenchyme of the nasopharyngeal epithelial folds that form during palatal shelf reorientation from vertical to horizontal position (Gkantidis et al., 2012; Figure 4A).

At ED13 and ED14, the secreted inhibitors, TIMP-1 and TIMP-2, display a similar spatial distribution to the MMPs and are widely expressed in the epithelial basement membrane. TIMP-3 is strongly expressed in the palatal epithelium although weakly expressed in the medial mesenchyme (Morris-Wiman et al., 2000a). Of the four TIMPs, TIMP-2 is the most abundant (Mansell et al., 2000), whereas TIMP-3 has different roles than the other TIMPs. Animals lacking the *Timp3* gene develop several pathologies associated with increased ECM degradation and loss of tissue integrity due to unregulated MMP, ADAM, and ADAMTS activity (Sahebjam et al., 2007). The anchored-membrane MMP inhibitor, RECK, is also expressed in the mesenchyme (de Oliveira Demarchi et al., 2010). RECK expression has been implicated in tissue integrity since its absence leads to extensive disarrangement of the connective tissue and embryos die *in utero* before craniofacial development (Oh et al., 2001). Expression of TIMP-3 and RECK in the different sites of the developing palatal epithelium suggests that they function in the maintenance of palatal tissue integrity by regulating epithelial–mesenchymal interactions (Figure 4B).

Matrix metalloproteinases are fundamental for the removal of the basement membrane and are expressed by epithelial cells in the EMT program to detach from MES and migrate to the adjacent mesenchyme to allow palatal fusion (Horejs, 2016). The participation of the membrane-anchored MMPs (MMPs -14, -16, and -25) appear to be crucial in this process (Shi et al., 2008; Brown and Nazarali, 2010). These observations are reinforced by the results of *in vitro* studies showing that addition of synthetic MMP inhibitors (Blavier et al., 2001), or the silencing of MMP genes (e.g., MMP-25) (Brown and Nazarali, 2010) in palatal cultures can prevent palatal fusion. Furthermore, *Tgfβ-3*- and *Egfr*-knockout mice, which display a cleft palate phenotype, have decreased or absent MMP expression in the MEE or MES (Miettinen et al., 1999; Blavier et al., 2001). Heparanase has also been detected in the MEE and MES and co-localized with MMPs -2, -3, and -9 (Hirata et al., 2013). MMP-25 can cleave only collagen IV and, in terms of substrates, displays more similarities to MMP-3 than other MT-MMPs (English et al., 2001). High gelatinolytic activity and laminin expression have also been found in the MEE and MES (Gkantidis et al., 2012). Furthermore, MMP-3 cleaves E-cadherin (Lochter et al., 1997) and MMP-25 is co-localized with E-cadherin in cell–cell junctions (Radichev et al., 2010), a crucial step for epithelial EMT cells to acquire a mesenchymal phenotype. Taken together, the basement membrane and epithelial cell–cell junction degradation require the cooperative proteolytic actions of MMPs and other ECM degrading enzymes in the MEE and MES cells (Figures 4C,D).

TGFβ-3 is expressed explicity in the palatal epithelium, and is co-expressed with MMPs (Blavier et al., 2001; Brown and Nazarali, 2010). In *Tgfβ-3* null mice, TIMP-2 and MMP-13 expression in the palatal epithelia were significantly decreased, whereas no changes in expression were noted for MMP-14 (Blavier et al., 2001). Similarly, the incubation of palatal culture with a TGFβ-3-neutralizing antibody decreases MMP-25 expression in the palatal epithelia (Brown and Nazarali, 2010). Collectively, these observations suggest that both MMP-13 and MMP-25 are downstream targets of TGFβ-3 (Figure 4C). MMP-13 is specifically expressed in sites of bone formation *in vivo* (Fuller and Chambers, 1995; Gack et al., 1995; Mattot et al., 1995; Stahle-Backdahl et al., 1997), and in physiological situations that require rapid and effective remodeling of collagenous ECM.

While MMP and TIMP expressions appear to be critical throughout all stages of palatal development, knockout mice for *Timp1, Timp2*, *Timp3, Timp4, Mmp2*, *Mmp9*, *Mmp13*, *Mmp14*, *Mmp16*, and *Mmp25* do not develop cleft palate (Paiva and Granjeiro, 2014; Soria-Valles et al., 2016). Interestingly, combined double knockout of *Mmp14* and *Mmp16* in mice leads to severe structural and craniofacial defects, including severe dysfunction in palatal shelf formation and cleft palate in 80% of the embryos (Shi et al., 2008). Among the membrane-anchored MMPs, MMP-16 is closely related to MMP-14 in terms of molecular structure and expression patterns in remodeling tissues (Szabova et al., 2005; Shi et al., 2008). While these findings suggest that potential compensatory mechanisms exist to overcome the loss of function of individual MMP genes, they also suggest that the loss of specific MMPs, in combination, may impair embryonic and palate development. Moreover, while individual MMP genes may not contribute a major gene effect to non-syndromic CL/P (NSCLP) susceptibility, they may act as modifiers on the background of other genes.

## Cleft LIP And/Or Palate

Orofacial clefts result from the failure of developing embryonic facial and palatal processes to ultimately merge or fuse. A multidisciplinary team including surgical, dental, speech, genetic, and nutrition experts are typically involved in patient care to mitigate the feeding, swallowing, breathing, speech, and hearing complications inherent to the condition. Orofacial clefts can be categorized as syndromic or non-syndromic (also termed isolated), based on the presence of additional structural abnormalities. Over 500 syndromes, including chromosomal abnormalities, have been reported in association with orofacial clefts, comprising 30% of all cleft cases. The remaining 70% of cases are all non-syndromic (i.e., isolated), and may segregate in families or appear in sporadic cases (Mossey et al., 2017).

**FIGURE 5:**
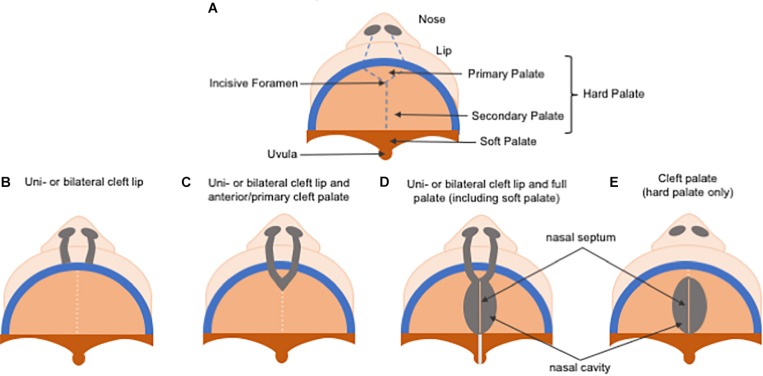
Schematic representation of cleft lip and/or palate classification. **(A)** Normal palate. **(B)** In uni or bilateral cleft lip, only lip reconstruction is necessary. **(C)** In uni or bilateral cleft lip and anterior/primary cleft palate, repair of the alveolar bone repair in the primary hard palate (pre-maxilla) is also required. **(D)** In uni or bilateral complete cleft lip and palate, treatment is the most challenging due to the need to repair both primary and secondary palate, and the soft palate is affected as well. **(E)** A cleft palate, which only comprises the secondary palate.

Non-syndromic orofacial clefts are the most common of congenital craniofacial disabilities, affecting approximately 1 in 700 live births worldwide each year, and have been divided historically into cleft lip, with or without cleft palate (CL/P), and cleft palate only (CP), due to the distinct developmental origins of the lip and the palate (Figure 5). While their reported prevalences vary considerably according to ancestral origin, Asians are the most frequently affected population, with birth prevalence rates as high as 1 in 500 live births, followed by Caucasians with a prevalence rate of about 1 in 1000 live births, and African populations showing the lowest prevalence rates at approximately 1 in 2500 live births (Kadir et al., 2017; Mossey et al., 2017; Ishorst et al., 2018). The presence of CL/P also differs by sex and laterality, with CL/P being more common in males than females at a 2:1 ratio, and CP being more common in females, meanwhile unilateral left CL/P is more common than unilateral right CL/P at a 2:1 ratio.

The etiology of non-syndromic orofacial clefts is complex, with both genetic and environmental factors contributing to the condition. While the identification of the genes involved in the syndromic forms of clefting has been mostly successful, much remains to be learned about the factors involved in non-syndromic cases. Genetic studies to date, using both family-based and case-control research approaches, have identified several genes and loci that may play a role in susceptibility to oral clefts (Vieira, 2008). Of these, the evidence is particularly strong for MSX1, IRF6, FOXE1, MAFB, WNT3, WNT9B, CRISPLD2, FGFR1, FGF8, BMP4, and the 8q24 chromosome region. Additional variants in numerous other genes (e.g., *TGF-β3*, *TGF-α*, *MMP3*, *VAX1*, *ABCA4*, *AXIN2*) have also been suggested as candidates for oral clefts with population-specific effects (Dixon et al., 2011; Beaty et al., 2016).

## ECM Remodeling Enzymes as Candidate Genes for Orofacial Clefts

Matrix metalloproteinases and TIMPs have been considered plausible candidate genes for CLP, based on their expression patterns in craniofacial tissues and their roles in tissue remodeling and morphogenesis during early embryogenesis; however, a functional role for any individual MMP or TIMP in palate development remains unknown (Brown et al., 2002; Verstappen and Von den Hoff, 2006).

**FIGURE 6:**
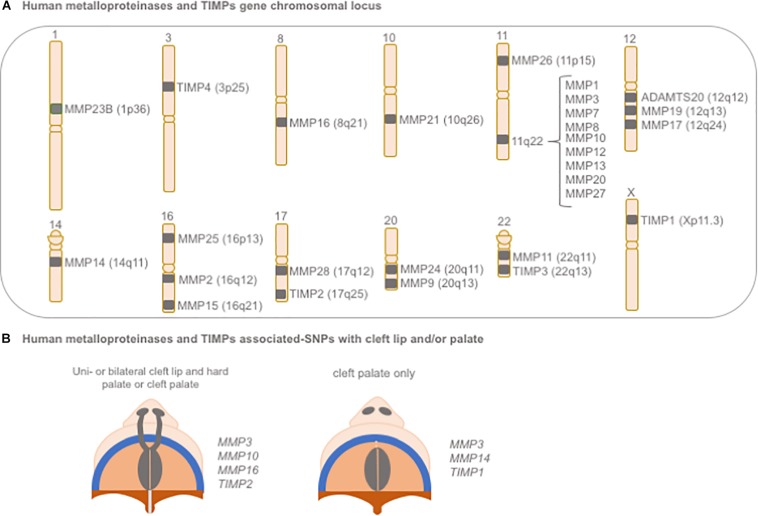
Schematic representation of **(A)** gene chromosomal locus of human metalloproteinases and TIMPs and **(B)** their associated-SNPs with cleft lip and/or palate. SNP: single nucleotide polymorphism.

In humans, significant associations between polymorphisms in MMP and TIMP genes with NSCLP have been reported (Blanton et al., 2004; Nikopensius et al., 2011; Letra et al., 2012; Figure 6). A large genome scan of multiplex NSCLP families first suggested evidence for linkage within a region on chromosome 16p13.3, in the same location for *MMP25* (Blanton et al., 2004). A later large and comprehensive study evaluated 45 polymorphisms spanning all biologically relevant MMP and TIMP genes for their association with NSCLP; significant associations (*P* < 0.0004) were noted for variants in *MMP3* (rs3025058, rs522616) and *TIMP2* (rs8179096), when considering CL/P, CP, and all cleft cases combined. Additional nominal associations were also found for variants in *MMP16* (rs7828497, *P* = 0.01) and *MMP10* (rs17293607, *P* = 0.06) in CL/P and all cleft cases. For CP, variants in *MMP3*, *MMP14*, and *TIMP1* also showed nominal associations (*P* < 0.05). Of note, certain allele combinations (i.e., haplotypes) involving these MMP and TIMP gene variants were also significantly associated with NSCLP (Letra et al., 2012).

Two polymorphisms, -1171 5A/6A (rs3025058) and -709 A/G (rs522616), located in the *MMP3* gene promoter were significantly associated with NSCLP and shown to have functional effects on gene transcription and protein function. The 5A/6A polymorphism consists of a common adenine insertion/deletion polymorphism (5A/6A) at position -1171 of the gene promoter and modulates transcription and local expression of the MMP-3 protein. The 6A allele showed an approximately twofold lower amount of gene product, compared with the 5A allele (Ye et al., 1996), and this difference in promoter activity was attributed to a likely differential binding of the transcriptional repressor to the 6A allele (Mercapide et al., 2003).

A positive regulatory element has also been described for *MMP3* -709 A/G, for which significantly higher (∼3.4-fold) promoter activity was found in the presence of allele A. This suggested that allele A can enhance promoter activity, possibly by augmenting transcription factor binding. Furthermore, this variant also appears to be modulated by the concomitant occurrence of the -1171 5A/6A variant. When analyzing the transcriptional effects of haplotypes containing both the -1171 5A/6A and -709 A/G variants, a 1.5-fold decrease in activity was observed for the combination of 5A_A alleles, in comparison with the 5A_G haplotype. Although speculative, this finding may represent a negative feedback loop effect, in an attempt to limit transcription in the presence of the two “high transcription” alleles, 5A and A. In contrast, a fourfold increased activity was found with the 6A_G promoter. The 6A_A haplotype was the least active promoter, suggesting potential gene downregulation with this allelic combination (Letra et al., 2014). Taken together, these observations indicate that the -709 A/G variant may directly regulate *MMP3* promoter activity, although its function was shown to the driven by the 1171 5A/6A alleles in the background.

Polymorphisms in the *TIMP2* gene have also been associated with NSCLP in different populations. A promoter variant in *TIMP2* (-180C/T; rs8179096) was strongly associated with oral clefts in a Brazilian population, whereas additional variants of unknown significance (intronic) were associated with NSCLP in US and Northeastern European populations (Nikopensius et al., 2011; Letra et al., 2012). Furthermore, functional analysis suggests that this variant has distinct allele-dependent effects, with the T allele presenting a 2.5-fold increased promoter activity. Furthermore, both C and T alleles were found to be putative binding sites for NFκB, a key transcription factor involved in the innate immune system. While C and T alleles reduced binding capability when NFκB consensus binding oligo diverges from protein in the same reaction, introduction of a mutant NFκB immunized C and T alleles from binding abolition (Letra et al., 2014). Additional studies are still necessary to unveil the exact mechanisms by which MMPs and TIMPs might contribute to NSCLP; nonetheless, allelic polymorphisms in these genes and their interactions may partly explain the variance in individual susceptibility to oral clefts.

Few studies have described roles for other ECM remodeling or cross-link enzymes during palatogenesis and their potential association with CL/P. ADAMTS-9 and -20 have been shown to participate in versican remodeling during palatogenesis (Enomoto et al., 2010); furthermore, ADAMTS-9 null mice die *in utero* (Dubail and Apte, 2015). During physiological palate formation, ADAMTS9 expression in the palatal shelves was restricted to microvascular endothelial cells, derived from the mesoderm, whereas CNC-derived mesenchymal cells express ADAMTS20; in contrast, the expressions of ADAMTSs 4 and 5 were not detected during palatogenesis (Enomoto et al., 2010). More recently, a genome-wide association study in both dogs and humans independently identified *ADAMTS20* as a potential candidate gene for clefting. A region on chromosome 27 in dogs was found to segregate with a complex phenotype of cleft palate and syndactyly; follow-up whole genome sequencing studies then identified a frameshift mutation in the *ADAMTS20* gene as potentially etiologic. Furthermore, four novel risk variants in *ADAMTS20* were identified in Guatemalan cases with NSCLP (Wolf et al., 2015).

Additional genes with roles in collagen maturation and other ECM molecules have also been suggested to play a role in NSCLP. The *LOXL3* gene located on chromosome 2p13.1 and mutations in this gene have been reported in association with Stickler syndrome (MIM #108300), an autosomal recessive disorder characterized by ocular, auditory, skeletal, and orofacial abnormalities (Alzahrani et al., 2015). In mice, the deletion of *Loxl3* causes neonatal mortality due to impaired collagen assembly and cross-linking, as well as spine deformity and cleft palate (Zhang et al., 2015). Furthermore, its ortholog in zebrafish is required for adequate craniofacial morphogenesis, as its loss of function results in abnormal chondrogenesis with micrognathia or agnathia phenotype (van Boxtel et al., 2011). Recently, a missense mutation in *LOXL3* (rs17010021; p.Ile615Phe) was reported to contribute to an increased risk of NSCLP (10-fold) in a European population (Khan et al., 2018).

## New Approaches to Studying Palatogenesis and Cl/P *In Vitro*

**FIGURE 7:**
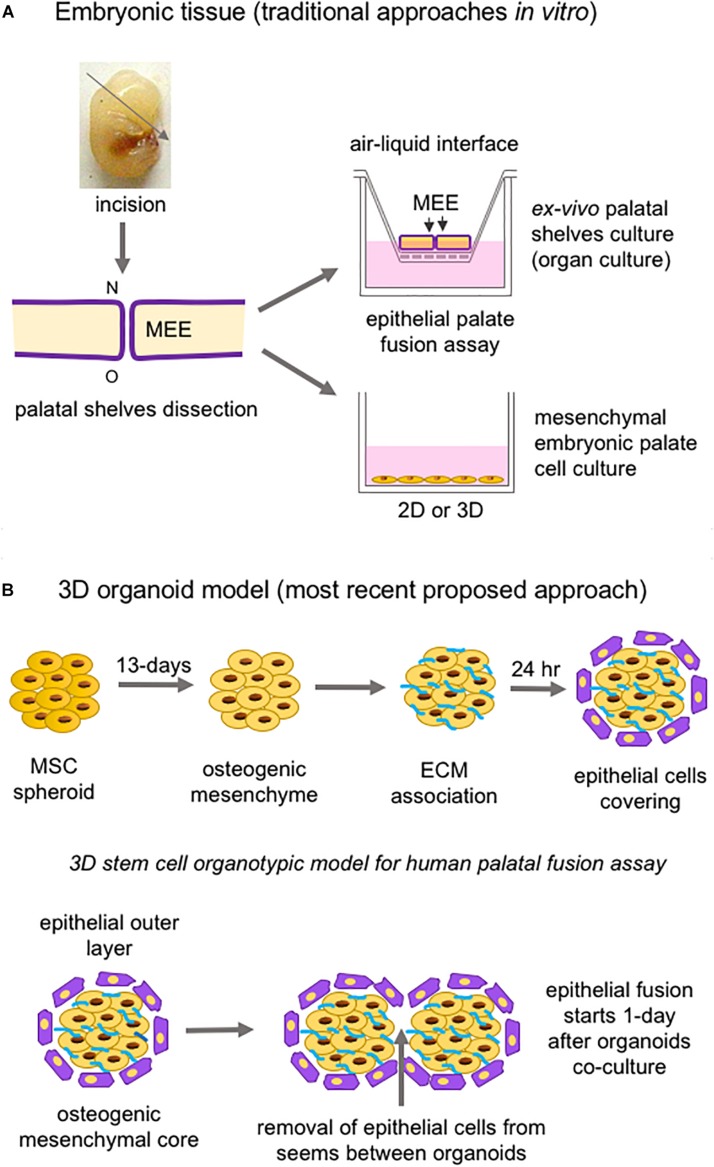
Schematic representation of *in vitro* approaches to study palatogenesis. **(A)** The most traditional method is the mouse embryonic palate dissection before palatal shelves fusion; the dissected palate is placed on a membrane insert, and the top part is in an air-liquid interface (organ culture); also, mesenchymal embryonic palate cells are cultivated under adhesion and monolayer on or not embedded into a substrate. **(B)** The newest proposed 3D organoid model using mesenchymal stem cells and epithelial cells to form spheroids/organoids to mimetize the epithelial fusion. ECM: extracellular matrix; MEE: medial edge epithelia; MSC: mesenchymal stem cell; N: nasal site; o: oral region; O: oral site.

Studies of both palatogenesis and CL/P are usually carried out in animal models (mouse, chicken, zebrafish, and rat, respectively), especially mice, given the similarity of biological events in these animals to humans (Van Otterloo et al., 2016; Lough et al., 2017). *In vitro*, the most common method of study employs murine embryonic palatal cell culture and *ex vivo* palate organ culture (Figure 7A; Abbott, 2019).

**FIGURE 8:**
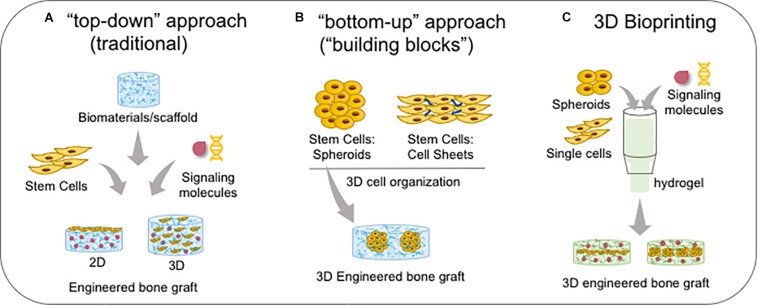
Bone bioengineering based-techniques. **(A)** Traditional “top-down” approach which stem cells are associated with biomaterials and signalling molecules to create a 2D (when single cells are seeded onto the substrate/biomaterial) or 3D microenvironment (when single cells are seeded into the substrate/biomaterial). **(B)** The newest “bottom-up” approach which cells are self-assembled in a 3D conformation by cell-cell and cell-ECM interactions as spheroids or cell sheets, forming the functional “building blocks”; spheroids can be embedded into scaffolds/biomaterials. **(C)** Both sing cells and spheroids and/or signalling molecules are the components of bioink in association with a hydrogel, and then it is bioprinted in a specific 3D engineered bone graft.

Three-dimensional (3D) cell culture represents a promising approach to better elucidate cell behavior, ECM remodeling, tissue remodeling, and tissue fusion, and ultimately to investigate clinical applications for tissue engineering (Ong et al., 2017). 3D constructs are more similar to the tissue microenvironment than classical 2D cell cultures (plastic-based) as they demonstrate more realistic cell morphology and physiology; furthermore, observation of 3D cultures over time is considered to represent 4D systems (Bissell, 2017). Thus, over the last decade, numerous reviews have illustrated the switch from 2D to 3D cell cultures. Several methods to create 3D structures have been described, such as cell aggregates, spheroids or organoids (Alhaque et al., 2018), seeded onto decellularized matrices (Taylor et al., 2018), and cell sheets (Kim et al., 2019). Among these, cell spheroid is a technology in which cells are cultured in suspension to create a 3D structure using cell–cell interactions and “scaffold-free” strategy (Figure 8B). This technique yields detailed information regarding ECM remodeling through “omics” analysis since the generation, growth, and fusion of cells allow accurate monitoring (Fan et al., 2018; Schnellmann et al., 2018; Wong et al., 2018). Understanding the ECM and its soluble factors are essential to comprehend embryonic development and tissue repair and will contribute to the discovery of new therapeutic tools. Proteomics is an appropriate strategy to characterize ECM components under physiological and pathological conditions. A significant challenge for studying the ECM constitutes its solubilization and protein recovery. An optimized protocol developed by Naba et al. (2017) permitted the digestion of proteins into peptides that could be analyzed by mass spectroscopy; web tools were allowed the annotation and relative quantitation of the ECM proteins. These protocols allow a faster analysis of differentially expressed proteins in the ECM, eliciting the identification of biomarkers and therapeutic targets.

Stem cells from many tissue origins, especially MSCs, have been widely employed in 3D constructions due to their effortless isolation from various tissues of the body, potential for differentiation into mesenchymal and non-mesenchymal lineages, and therapeutic use (Han et al., 2019). Cell spheroids to study epithelial palate fusion are available, and strategies employed consist of creating mesenchymal spheroids from human umbilical-derived MSCs undergoing osteogenic differentiation, covered with ECM to mimic basement membrane, which are then seeded with human progenitor epithelial keratinocytes. This 3D organotypic model of human palatal shelves can maintain cell viability for around 4 weeks, expresses alkaline phosphatase, and is responsive to EGF, leading to increased epithelial proliferation and the prevention of epithelial fusion between spheroids (Figure 7B; Wolf et al., 2018). A potential bias relies on the fact that the MSCs used in the spheroid generation do not have the same embryonic origin as the palatal mesenchyme or the epithelial cells.

## State-Of-The-Art of Cleft Palate Repair and Palatal Reconstruction

### Bone Regeneration

**FIGURE 9:**
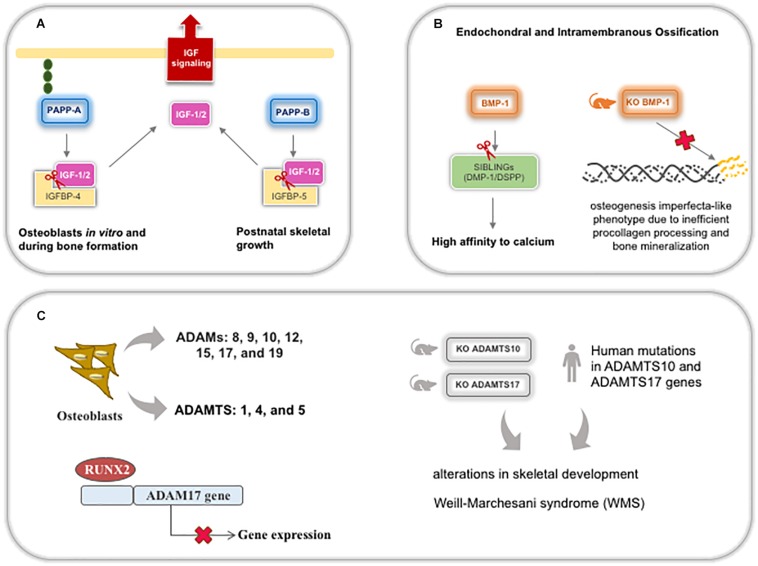
Schematic representation of metalloproteinases (except MMPs) expression and roles during bone development and postnatal skeletal growth. **(A)** PPAPP-A and B cleavage the complex IGFBP/IGFs to bioavailable IGF for bone cells. **(B)** BMP-1 is important for SIBLINGs cleavage, specifically DMP-1 and DSPP, leading these glycoproteins to increase their affinity to calcium and contributing for mineralization. **(C)** Several ADAMs and ADAMTSs are expressed by osteoblasts, being ADAM17 downregulated by RUNX2 during osteoblastic differentiation. Both ADAMTS10 and 17 knockout mice and human mutations display the same bone phenotype recognized in Weill-Marchesani syndrome. ADAM: adisintegrin and metalloproteinase; ADAMTS: adisintegrin and metalloproteinase with thrombospondin motifs; DMP-1: dentin matrix acidic phosphoprotein; DSPP: dentin sialophosphoprotein; IGFBP: insulin-like growth factor binding protein; IGF: insulin-like growth factor; KO: knockout mice; PAPP: pregnancy-associated plasma protein.

Bone repair is a mechanism in which bone development program is recapitulated to form new intramembranous or endochondral bone at injured sites. Intense ECM bone remodeling by MMPs is required (Paiva and Granjeiro, 2017) as well as the action of several growth factors (TGF-β, BMPs, FGFs, IGFs, PDGFs, and VEGFs), which are implicated in driving osteochondral differentiation, regulation of bone formation, and are responsible for triggering intracellular pathways, such as Wnt (Houschyar et al., 2019). Additionally, other metalloproteinases are important for collagen maturation and mineralization. PPAP-A increases osteoblast proliferation *in vitro* and bone formation *in vivo* by augmenting IGF bioavailability upon cleavage of IGFBP-4 (Qin et al., 2006). PPAP-B expression was associated with postnatal skeletal growth, bone mass, and structure due to cleavage of IGFBP-5, the most abundant IGF in bone (Figure 9A; Amiri and Christians, 2015; Christians et al., 2019).

In bone and dentin matrices, BTPs can cleave the acidic domain of SIBLINGs (small integrin-binding ligand, N-linked glycoproteins), specifically DMP-1 and DSPP (dentin sialophosphoprotein—generating DPP/dentin phosphoprotein), leading to an increase in the binding affinity of these proteins for calcium, which is necessary for mineralization and improving ECM stiffness (Steiglitz et al., 2004; von Marschall and Fisher, 2010; Tsuchiya et al., 2011). BMP-1 is highly expressed in both endochondral and intramembranous ossification sites during development and contributes to an increase in bone repair (Grgurevic et al., 2011). In addition, *BMP-1* expression is modulated by mutations in both alpha procollagen chains (Lindahl et al., 2011) and BMP-1-knockout mice develop an osteogenesis imperfecta-like phenotype due to inefficient procollagen processing and bone mineralization (Figure 9B; Muir et al., 2014). Although meprins also act on procollagen maturation and DSPP cleavage, meprin-β is inhibited in tissues with high concentrations of calcium and is, probably, not crucial in hard tissues since mice knocked out for meprins do not demonstrate alterations in bone and tooth development (Arnold et al., 2017).

Several ADAMs (8, 9, 10, 12, 15, 17, and 19) and ADAMTS (1, 4, and 5) are secreted by osteoblasts and bone tissue. Recently, ADAM17 has been reported to be a target of RUNX2 during osteoblastic differentiation, where ADAM17 is suppressed by RUNX2 (Araya et al., 2018). Human mutations in *ADAMTS10* and *17* are associated with related syndromes involved in alterations in skeletal development (Dagoneau et al., 2004; Morales et al., 2009). Recently, knockout mice for both *Adamts10* and *17* were shown to develop the recapitulate syndromic phenotype in human (Figure 9C; Mularczyk et al., 2018; Oichi et al., 2019). Reduced hypertrophic zone and increased deposition of fibrillin-2 alter the growth plates during endochondral ossification resulting in adults with short statures. Fibrillins are glycoproteins involved in microfibril formation and elastin deposition. Also, treatment with BMP-2 can rescue terminal chondrocyte differentiation, suggesting that ADAMTS17 is important for ossification through the modulation of BMP signaling (Oichi et al., 2019).

Extracellular matrix cross-linkers also contribute to bone formation and regeneration. In LOX knockout mice, general ECM architecture is profoundly affected in several tissues, and littermates die soon after birth. In bone, early and late osteoblastic differentiation and onset of mineralization are decreased in these mice as well as the gene expression of LOX isoforms (LOXL1-4) and osteoblastic markers (collagen type I, bone sialoprotein, and RUNX2) (Pischon et al., 2004).

The crosstalk between inflammatory cells, MSCs, chondrocytes, osteoblasts, osteoclasts, and osteocytes is necessary (Liu et al., 2018), and the success of bone regeneration results from the balance of the osteoprotegerin/RANK/RANKL axis. Most bone defects regenerate spontaneously; however, extensive bone loss due to trauma or aging-related fractures, metabolic bone disease, and congenital malformations do not regenerate *per se*, underscoring the search for better drug candidates. Biomaterials, biomolecules, and stem cells have been investigated to support bone repair, overcoming the limitations of autologous and allogeneic grafts (Collignon et al., 2017).

### First Generation Palatal Reconstruction: Standard Surgical CL/P Management and Cleft Palate Repair

**FIGURE 10:**
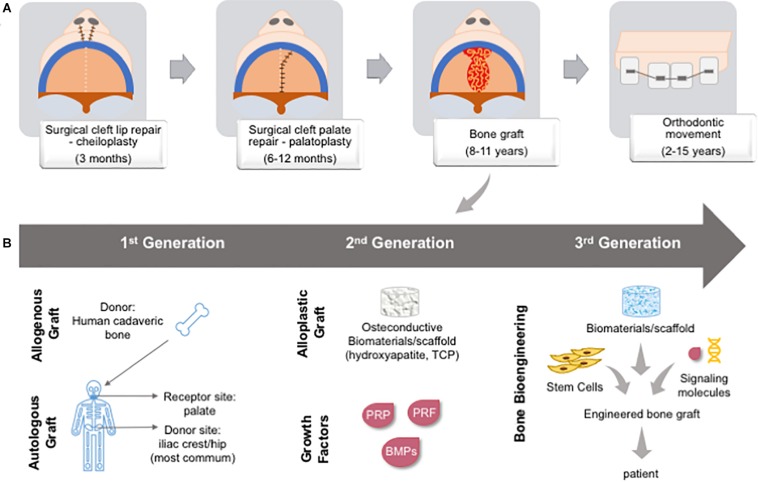
Cleft lip and/or palate patient chronological management over time and types of bone graft. **(A)** First, patients are submitted to a lip reconstruction (around 3 months); followed by palate reconstruction (between 6–12 months); primary bone graft in the hard palate (between 8–11 years); and, then orthodontic movement (between 2–15 years). **(B)** Bone graft was developing from gold-standard autologous and allogenous graft (1^st^ generation), the development of osteoconductive biomaterials and recombinant growth factors or natural adjuvants (2^nd^ generation), and bone bioengineering is bringing a new possibility to associate stem cells from patient, biomaterials and signaling molecules to create an *in vitro* engineered bone graft to be implanted in the patient, being a personalized approach (3^rd^ generation). BMP: bone morphogenetic protein; PRF: platelet-rich fibrin; PRP: platelet-rich plasma; TCP: tricalcium phosphate.

The treatment of CL/P patients is primarily surgical (plastic surgery for the lip and a bone graft for the palate) followed by orthodontic treatment in multiple stages over the years. The management is dependent on the cleft site, extension, and affected tissues (Farronato et al., 2014). The term “palatal reconstruction” is used to define any intervention able to restore the barrier between the oral and nasal cavities, and physiological functions. As such, several approaches, such as the lip and/or palate closure through plastic surgery, several types of bone graft, or a combination of these have been employed. More recently, the use of bioengineered bone has allowed palatal reconstruction (Figure 10). The decision with regard to the best treatment of choice depends on the type of CL/P and the extension of the tissue loss (Gupta et al., 2012).

Autogenous bone grafts are the gold standard for alveolar bone and cleft palate repairs. Throughout treatment stages, surgical procedures may require several grafts (e.g., primary, secondary, and tertiary graft). The most common bone donor sites are the anterior iliac crest, proximal tibia, mandibular symphysis, calvaria, and ribs. However, the bone amount and volume available from these sites are restricted, and a most advantageous site is still under debate. The use of autogenous bone graft has many drawbacks related to morbidity at the donor site, and patients may present chronic pain at the site, paresthesia of the thigh, and hypertrophic scar. The loss of the graft due to local inflammation, bone resorption, and the development of oronasal fistulas is also frequently associated with unsuccessful repair (Borba et al., 2014; Tavakolinejad et al., 2014; Shafi et al., 2015).

### Second Generation of Palatal Reconstruction: Biomaterials and Growth Factors

Bone graft options available, such as allogeneic bone (Shirani et al., 2017), xenogeneic bone (Thuaksuban et al., 2010), and alloplastic graft (Kumar et al., 2013; Seifeldin, 2016; Sharif et al., 2016), still do not promote effective palatal reconstruction, stimulating the search for better alternatives. To overcome the limitations of autologous and allogeneic bone grafts, studies have investigated the use of the association, or not, of osteoconductive biomaterials, such as hydroxyapatite and tricalcium phosphate, with autologous bone graft or growth factors, such as BMP, which is known to stimulate bone formation and repair (Horch et al., 2006; Weijs et al., 2010; Lazarou et al., 2011; Benlidayi et al., 2012; De Ruiter et al., 2015; Takemaru et al., 2016; Martín-Del-Campo et al., 2019). After the systematic review of 29 studies, just two were used for qualitative and quantitative analysis (Segura-Castillo et al., 2005; Dickinson et al., 2008). However, considering some aspects of randomization, allocation concealment, blinding, outcome data, and reporting, the authors concluded that the two selected articles presented a high risk of bias and that conclusions concerning the benefits of the interventions, compared to traditional iliac grafting, could not be made (Guo et al., 2011).

Growth factors such as BMPs (2, 4, and 7) and “natural adjuvant” platelet concentrates, denominated platelet-rich plasma (PRP) and platelet-rich fibrin (PRF), have been employed in palate reconstruction (Behnia et al., 2012; Seifeldin, 2016). Interestingly, PRF does not affect the expression of the RUNX2, BMP-2, or RANKL, but induces the expression of OPG, leading to the increase of OPG/RANKL, suggesting that PRF could boost osteoblastic differentiation (Sumida et al., 2019).

Clinical studies of alveolar bone repair in CL/P patients tested the association of BMPs or platelet concentrates with the gold standard of autologous bone graft. BMPs make relevant contributions to bone embryogenesis and repair (Salazar et al., 2016) and the inhibition of BMP receptors (Lai et al., 2016), mutations (Sahoo et al., 2011), and gene polymorphism (Savitha et al., 2015) are involved in cleft development. The association of bone graft with human recombinant BMP-2 (hrBMP-2) *in vitro* and *in vivo* demonstrated the production of mature bone (Shimakura et al., 2003). Furthermore, clinical studies corroborate that BMPs are, at least, just as efficient as autologous bone graft for the repair of alveolar/palate cleft (Chin et al., 2005; Canan et al., 2012; Ayoub et al., 2016; Hammoudeh et al., 2017). Very recently, 10 years of follow-up evidenced the safe use of BMP-7 for the reconstruction of the alveolar cleft (Ayoub and Gillgrass, 2019).

Evaluating the use of growth factors for the reconstruction of the alveolar cleft, Van Hout group accessed 291 studies using BMP-2, BMP-7, TGF-β, PDGF, IGF, FGF, VEGF, and PRP (van Hout et al., 2011). Only six articles met the authors’ criteria for full analysis, who concluded that BMP-2 improved the quantity of bone formation during the reconstruction of the alveolar cleft, in association with reducing pain at the donor site, a reduction in surgery time, hospital stay, and overall cost. A recent study reported a similar effectiveness of autologous bone and rh-BMP2 during the maxillary reconstruction of cleft lip and palate patients (Scalzone et al., 2019).

A growing field of investigation aims to evaluate the use of cell-based therapies to treat alveolar and palate cleft (Gładysz and Hozyasz, 2015; Brozek et al., 2018) due to the ability of stem cells to differentiate into active osteoblasts that drive bone regeneration. Trabecular bone was enhanced after the treatment of 16 out of 17 alveolar clefts with MSCs together with hrBMP-2, but long-term follow-up studies are still needed (Fallucco and Carstens, 2009).

Two recent systematic reviews addressed the role of BMP2 (Scalzone et al., 2019) and tissue-engineered bone replacement materials (Kamal et al., 2018) in the efficient treatment of palate and alveolar cleft. Both studies concluded that there was no statistical difference between the autologous bone graft and the alternative materials. The meta-analysis comparing the bone repair with iliac crest bone graft (ICBG) versus BMP-2, acellular dermis matrix membrane, and cranium or rib grafts, indicated that ICBG is still the best choice for treatment (Wu C. et al., 2018). The association of BMP-2 with a collagen sponge provides similar results to those of ICBG, and PRP associated with ICBG increased bone retention for skeletally mature patients. However, better-designed controlled studies are required for long-term analysis of alveolar cleft reconstruction, with follow-ups of greater than 12 months (Seifeldin, 2016; Liang et al., 2018; Wu C. et al., 2018). Furthermore, the development of a consensus for standardized protocols, using multicenter studies, is still needed (de Ladeira and Alonso, 2012).

### Third Generation of Palatal Reconstruction: Bone Bioengineering

Tissue bioengineering represents a new therapeutic alternative for palatal reconstruction, associating the use of stem cells, biomaterials/scaffolds, and signaling molecules. Two main approaches are dominant: the “top-down” approach, in which classically cells are seeded on or into biomaterials for recreating a new 3D tissue *in vitro*; and the “bottom-up” approach, which uses 3D construction techniques (described in the last section) to improve cell secretion of growth factors for tissue regeneration *in vivo* (Figure 8; Baptista et al., 2018). Furthermore, Good Manufacturing Practice (GMP) and animal-free supplements are crucial for clinical applications. The success of the bioengineered bone graft is directly related to the osteogenic potential of stem cells, biomaterial/scaffold specific properties, and adequate external signaling from growth factors.

Stem cells from different origins and potentialities represent the cutting-edge technology for palatal reconstruction (Zuk, 2008; Gładysz and Hozyasz, 2015). However, few studies of bone bioengineering approaches in animal models of palatal defects have been carried out. A large number of research articles have addressed bone bioengineering approaches for alveolar bone defects created in animal models to reproduce alveolar cleft or mid-palate cleft. However, here we consider only animal models of the mid-palate cleft and the use of human cells. Conejero et al. (2006) demonstrated that rat palatal critical-size defect filled with autogenous engineered graft (fat-derived stem cells previously differentiated into osteoblasts/osteocytes and seeded onto poly-L-lactic acid absorbable scaffolds) could regenerate bone at 6 or 12 weeks after surgery; bone defects filled only with the scaffold or scaffold plus undifferentiated cells had a fibrotic tissue with no bone. Another study used an autogenous multi-layered graft, which was simultaneously bioengineered for palate bone and oral mucosa in a rabbit palatal critical-size defect (Martín-Piedra et al., 2016). Initially, individual cell layers of adipose tissue-derived MSCs were seeded onto fibrin-agarose hydrogels and induced toward osteogenic differentiation; and with fibroblasts and keratinocytes seeded onto fibrin-agarose hydrogels, maintained in epithelial culture medium and under air–liquid conditions. Subsequently, the oral mucosa layer was placed on the top of the osteogenic layer and compressed to induce nanostructured fusion of the mucosal stroma (fibroblasts) and the osteogenic layer. The 3D multi-layered graft was able to integrate with host tissue, and achieved partial bone differentiation; the authors suggested that complex multi-layer constructions could increase the maturation times compared to monolayers *in vivo*.

Bone-marrow stem cells (BMSCs) are the “gold standard” for several cell- and tissue-based clinical applications. These are, to date, the most studied stem cells and their properties are well known, but it is not clear whether the application of BMSCs or other MSCs in craniofacial bone regeneration requires handling *ex vivo* and/or pre-differentiation before clinical application (Shanbhag et al., 2019). For alveolar cleft repair in CL/P patients, scaffold-free BMSCs are safe, but this material is not suitable for extensive bone defect repair (Bajestan et al., 2017). The associations of BMSCs with commercial demineralized bone matrix (Osteoset DBM) (Behnia et al., 2009), tricalcium phosphate (Du et al., 2017), platelet-derived growth factor on biphasic hydroxyapatite/tricalcium phosphate (Behnia et al., 2012), or PRF membrane (Mossaad et al., 2019), have been shown to contribute to bone repair. However, the use of BMSC does not reduce the morbidity caused by iliac crest donor site handling even when using minimally invasive techniques. Thus, other sources of MSCs are necessary to eliminate this side effect.

The MSCs found in adult dental tissues display cranial NCC properties (Liu and Cheung, 2016; Niibe et al., 2017; Cui et al., 2018), as these embryonic cells are more similar to palate forming cells than BMSCs. In the oral cavity, human MSCs have been isolated and characterized from odontogenic and non-odontogenic origins, permitting the harvesting of healthy tissues during dental surgical procedures. Thus, a subset of cells displaying MSC properties and osteogenic properties have been described from gingival connective tissue [gingival mesenchymal stem/progenitor cells (GMSCs)] (Yang et al., 2013), oral periosteum of the palate (Caballero et al., 2010; Ceccarelli et al., 2016), the lower and upper vestibule (Ceccarelli et al., 2016), palatal connective tissue (Roman et al., 2013; Páll et al., 2015, Pall et al., 2017), and adipose stem cells from buccal fat pad (Farré-Guasch et al., 2010). Recently, palatal periosteum-derived MSCs cells, cultivated under serum- and xeno-free conditions, and cells were able to retain stem cell properties (Naung et al., 2019). One registered clinical trial was conducted using adipose stem cells from the buccal fat pad (Khojasteh et al., 2017), associated with ICBG, lateral ramus cortical bone plate, and bovine mineral graft, with all groups producing statistically similar results. However, these reports presented a limited source of tissue, and were assessed in specific situations, and therefore more effective cell sources are still required.

Five MSC populations are found in dental tissues: (I) in the dental follicle [dental follicle progenitor stem cells (DFPSCs)]; (II) in the apical papilla [stem cells from apical papilla (SCAPs)]; (III) in the ligament [periodontal ligament stem cells (PDLSCs)]; (IV) in the adult dental pulp [dental pulp stem cells (DPSCs)]; and (V) in the dental pulp of deciduous teeth [stem cells from exfoliated deciduous teeth (SHEDs)] (Baniebrahimi et al., 2018). Of these, SHEDs, which have exfoliative characteristics, are the most easily obtained odontogenic tissue, via a little or non-invasive procedure. The pulp tissue can be obtained during the period of the changing of the child’s teeth, between 5 and 12 years of age, with insignificant ethical implications and provides a suitable alternative for pediatric regenerative medicine (Taguchi et al., 2019). SHEDs display high proliferative capacity, multi-lineage differentiation, secretion of immunomodulatory molecules. DPSCs, similarly to SHEDs, could be an alternative source of cells from teenagers or during adulthood during dental procedures being and are easily harvested from third molars routinely indicated to exodontia (Yamada et al., 2019). Both DPSCs and SHEDs allow cell sheets (Pedroni et al., 2018; Lee et al., 2019) and 3D spheroids cultures (Wang et al., 2010; Xiao et al., 2014). The high regenerative potential of SHEDs and DPSCs could be explained by its particular secretome content, including many types of paracrine soluble molecules and EVs, identified as immunogenic, pro-neurogenic, and pro-angiogenic (Kichenbrand et al., 2018; Marei and El Backly, 2018; Yusof et al., 2018). SHED secretome profile is also modulated during osteogenic differentiation leading to increase angiogenic potential (Mussano et al., 2018).

Concerning bone repair, SHEDs are better for forming new bone in a calvaria critical-size defect model, when compared to other dental MSCs and BMSCs. Recently, human SHEDs seeded onto dense collagen hydrogels, which were primed with FGF-2 and submitted to hypoxia conditions before implantation, improved intramembranous bone formation in an immunodeficient calvaria critical-size bone defect mouse model (Novais et al., 2019). Most of the 56 articles thoroughly analyzed in a systematic review reported good results and the relevance of human DPSCs for bone engineering in animal models or human clinical treatments (Leyendecker Junior et al., 2018). Similarly, a narrative review of 39 studies also concluded that DPSCs and SHEDs were of value for bone tissue regeneration (Cristaldi et al., 2018). Since 2005, many countries have started to collect and store healthy exfoliated teeth, creating biobanks, which are of low cost in comparison to umbilical cord banks (Campanella, 2018). Unfortunately, *ex vivo* manipulation of stem cells is still required, representing a challenge since this is a high cost and time-consuming procedure.

Little information is available regarding the use of MSCs harvested from CL/P patients. Bueno et al. (2011) isolated and characterized MSCs from *orbicularis oris* muscle (denominated by the authors as “cleft lip and palate muscle-derived stem cells”), usually discarded during the initial surgery repair (cheiloplasty) of CLP patients. These cells are able to express classical MSC cell surface proteins and differentiate into osteogenic, adipogenic, chondrogenic, and skeletal muscle cell lineages *in vitro*. The cells, when seeded onto collagen membranes, display the ability to repair bone in an immunocompromised rat critical-size cranial defect model. The analysis of DNA variants affecting the gene expression (cis-eQTLs) of cleft lip and palate muscle-derived stem cells allowed the discovery of a new susceptibility locus for NSCLP was discovered (rs1063588), coincident with the *MRPL53* gene (Masotti et al., 2017). Bueno et al. (2011) compared the gene expression profile of SHEDs from healthy donors and CL/P patients and verified that 87 genes presented differential expressions, with more than a half being glycoproteins related to the ECM (collagens, MMPs, integrins, and adhesion proteins). This study showed that MSCs might be a powerful tool for genetic and “omic” studies of CL/P. Later, the same group showed that low power laser therapy could enhance the osteogenic potential of DPSCs from CL/P patients (Pinheiro et al., 2017). These results support the need for the study of MSCs from CL/P patients to better understand cell behavior, ECM secretion, and remodeling, and employ this knowledge to drive new strategies based on bone bioengineering for palatal reconstruction.

Another growing field related to regenerative medicine that could be applied to palate reconstruction is bioprinting. Spheroids are potential building blocks in 3D bioprinting, in a large-scale process for bone and cartilage tissue production. Growing evidence shows that 3D spheroids formed from MSC present increased angiogenic and chemotactic signaling (Costa et al., 2017). 3D bioprinted cryogels, formed from chitosan (CS)/gelatin-based scaffolds for personalized palate reconstruction, have been designed by CL/P computed tomography data (Hixon et al., 2017). A 3D bioprinted bioresorbable scaffold (polycaprolactone—approved by the Korean Ministry of Food and Drug Safety for clinical use) seeded with autologous BMSCs from the iliac crest in the operation room, was implanted in a 10-year-old Korean boy with a history of previously repaired unilateral CL/P presenting a cleft alveolus and an oronasal fistula. At 6 months after transplantation, the new bone formed reached around 45% of the total defect volume, suggesting that this new technology could be a promising alternative (Ahn et al., 2018).

Three-dimensional additive manufacture allows the production of biomaterials/scaffolds used successfully for bone bioengineering have to be biocompatible, specific porosity, chemical and topographical characteristics and surface properties for osteoconduction, biomechanical properties, biodegradability, and radiolucency, to induce osteogenesis (osteoinductivity) and vascular ingrowth. In a new vision, biomaterials may trigger immunological host responses to stimulate tissue regeneration (Franz et al., 2011). As we have previously seen, the provisional matrix is secreted by embryonic palatal cells to drive palatogenesis. Among biopolymers used as biomaterials/scaffolds for bone bioengineering, collagen type I, CS, and HA are the most commonly employed. CS is a polysaccharide chitin-derived present in invertebrate exoskeletons which displays many promising characteristics such as biocompatibility, antibacterial activity, biodegradability, porosity, immunomodulatory properties, promotes cell adhesion, proliferation, migration, and ultimately enhances bone regeneration due to stimulation of osteoblast differentiation and mineralization (Farhadihosseinabadi et al., 2019).

As we have seen previously, LOX and TGs are crucial enzymes for collagen–collagen cross-links. These enzymes have been employed to create cross-links in collagen-based biomaterials (Fortunati et al., 2014; Cai et al., 2017). This modern approach can replace the traditional chemical method employing aldehydes, isocyanates, and carbodiimides. Although this method creates strong stable cross-links, undesirable consequences such as cytotoxicity, calcification, and foreign body response are well known for these chemicals (Sorushanova et al., 2019). Specifically in bone tissue, TG induces the oligomerization of SIBLINSs, and calcium-binding proteins (osteopontin and bone sialoprotein), which may drive mineral nucleation or calcium crystal growth (Forsprecher et al., 2009).

## Concluding Remarks

Although several MMPs and their inhibitors TIMPs are expressed during palatogenesis, the cell membrane-anchored MMPs appear to represent the principal pericellular collagenases (MMP-14 and MMP-16) and may be crucial for the development of CL/P. However, recent studies have demonstrated roles for other metalloproteinases, such as ADAMTS in this pathology. There seems to be a crucial compensatory effect between MMPs to ensure that the various stages of palate formation can occur. However, more studies should be carried out on other metalloproteinases to better understand the complexity of ECM remodeling, the generation of bioactive molecules, and the relationship between them.

Cleft therapy still is dependent on bone grafts, mainly of the ICBG, but new approaches have been under evaluation. Administration of BMP-2 and PDGF combined with bone graft is promising, but recent systematic reviews support the need for better-designed randomized controlled clinical trials with long-term follow-up (>12 months). The development of *in vitro* models using stem cells from CL/P patients may be an exciting approach for further studies of the biology of these cells as well as their potential use in new individualized therapeutic approaches for palatal reconstruction. 3D culture has been used to recapitulate the critical events of development, such as cell–cell interaction, differentiation, growth and cell fusion, which, coupled with “omics” analysis and computational biology, promote considerable advances in tissue remodeling and repair. Additionally, the emerging and exciting field of EVs may provide critical information via “omics” analyses to understand how this component of ECM contributes to the palatogenesis of CL/P.

The development of new biomaterials that simulate a provisional matrix during palatogenesis and provide the controlled release of growth factors may aid to improve new therapeutic approaches based on bone bioengineering. Emerging cell reprogramming or trans-differentiation technologies could provide other unusual sources of therapeutic cells. The former consists of differentiated adult cells, such as fibroblasts, that are forced to overexpress transcription factors that regulate pluripotency generating inducible pluripotent stem cells (iPSCs) displaying the same characteristics as embryonic stem cells (Spyrou et al., 2019). The second approach consists of the direct conversion of a fully differentiated cell type into another one; for example, the conversion of fibroblasts into osteoblasts (Cho and Ryoo, 2018). Furthermore, another possibility is to use EVs released by MSCs as potential cell-free tools for bone regeneration (Praveen Kumar et al., 2019). Finally, we now have a vast knowledge of craniofacial development, CL/P and CL/P treatment, associated with many advances in cell and material engineering technologies. So, multidisciplinary efforts must be made to achieve advances in the quality of life of CL/P patients.

## Author Contributions

KP contributed to the design, writing, and financial support of the manuscript. CM, PS, AL, and JG contributed to the design and writing of the manuscript.

## Conflict of Interest

The authors declare that the research was conducted in the absence of any commercial or financial relationships that could be construed as a potential conflict of interest.

## Abbreviations

ABCA4: ATP-binding cassette sub-family A (ABC1) member 4
ADAM: a disintegrin and metalloproteinase
ADAMST: a disintegrin and metalloproteinase with thrombospondin-like motifs
BMP: bone morphogenetic protein
BMSC: bone marrow stem cell
BTP: BMP-1/Tolloid-like proteases
CL/P: cleft and lip palate
CRISPLD2: cysteine-rich secretory protein LCCL domain-containing 2
CSPG: chondroitin sulfate proteoglycans
DFPSC: dental follicle progenitor stem cells
DMP-1: dentin matrix protein-1
DSPP: dentin sialophosphoprotein
DPP: dentin phosphoprotein
DPSC: dental pulp stem cell
ECM: extracellular matrix
ED: embryonic day
EGFR: epidermal growth factor receptor
EMT: epithelial-mesenchymal transition
EV: extracellular vesicles
FGF: fibrobast growth factor
FGFR: fibrobast growth factor receptor
FOXE1: forkhead box E1
GAG: glycosaminoglycans
GDF: growth differentiation factor
GH: growth hormone
GMP: good manufacturing practice
GMSC: gingival mesenchymal stem/progenitor cell
GPI: glycosyl-phosphatidyl-inositol
GW: gestational weeks
HA: hyaluronic acid
Has: hyaluronan synthase
Hh: hedgehog
IGFBPs: insulin-like growth factor binding proteins
IGF: insulin growth factor
IRF6: interferon regulatory factor 6
KO: knockout mice
LOX: lysyl oxidase
LOXL: lysyl oxidase-like
LTBPs: TGF- β binding proteins
MAFB: V-maf musculoaponeurotic fibrosarcoma oncogene homolog B
MEE: medial edge epithelia
MES: medial epithelial seam
MMP: matrix metalloproteinase
MSC, mesenchymal stem cell mTLD: tolloid
MTLL-1: tolloid-like 1
MSX1: Msh homeobox 1
MV: matrix vesicles
NCC: neural crest cells
NFkB: factor nuclear kappa B
NSCLP: nonsyndromic cleft and lip palate
OPG: osteoprotegerin
PAPP: pregnancy-associated plasma protein
PCPE: procollagen C-proteinase enhancer
PDGF: platelet-derived growth factor
PDLSC: periodontal ligament stem cell
PRF: platelet-rich fibrin
PRP: platelet-rich plasma
Rac1: Ras-related C3 botulinum toxin substrate 1
RANK: receptor activator of nuclear factor κ B
RANKL: receptor activator of nuclear factor κ B-ligand
SHED: stem cells from exfoliated deciduous teeth
SCAP: stem cells from apical papilla
SRLP: small rich-leucine proteoglycans
SIBLING: small integrin-binding ligand N-linked glycoprotein)
TG: transglutaminase
TGF-α: transforming growth factor alpha
TGF-β: transforming growth factor beta
VAX1: ventral anterior homeobox 1
VEGF: vascular endothelial growth factor.
